# Brain Abscess Due to Dental Sinusitis: A Case Report on Incomplete Infection Defense Associated With a Post-Fusion Linear Skull Fracture

**DOI:** 10.7759/cureus.43941

**Published:** 2023-08-22

**Authors:** Shinya Watanabe, Yasushi Shibata, Eiichi Ishikawa

**Affiliations:** 1 Department of Neurosurgery, Mito Kyodo General Hospital, Tsukuba University Hospital Mito Area Medical Education Center, Mito, JPN; 2 Department of Neurosurgery, Institute of Medicine, University of Tsukuba, Tsukuba, JPN; 3 Department of Neurology, Mito Kyodo General Hospital, Tsukuba University Hospital Mito Area Medical Education Center, Mito, JPN

**Keywords:** frontal sinus, odontitis, linear skull fracture, odontogenic sinusitis, brain abscess

## Abstract

Brain abscess is a pyogenic disease secondary to a bacterial, tuberculous, or fungal infection of the brain; thus, early detection and treatment are of crucial importance. Herein, we present a case of a brain abscess arising from dental sinusitis due to an incomplete infection defense mechanism linked to a post-fusion linear skull fracture. The patient initially presented with a persistent headache, which was diagnosed as frontal sinusitis. Consequently, antibiotic treatment was started. However, due to a refractory response to antibiotics, MRI was performed, which revealed a brain abscess in the frontal lobe adjacent to the right frontal sinus measuring 40 mm in diameter. This abscess was surgically drained and cultured. Initially, the patient was treated with three antibiotics, which were eventually de-escalated. The cultures revealed nasal commensal bacteria, suggesting a direct spillover from sinusitis leading to a brain abscess. A tooth with root inflammation, which had been left untreated and resulted in bone melting of the maxillary sinus wall, was extracted. After more than eight weeks of antimicrobial therapy, improvement in the clinical and imaging findings was noted, and the patient was discharged. Brain abscesses may develop from sinusitis even after linear fractures have healed due to a continued incomplete infection defense mechanism. Moreover, root and sinus infections should undergo evaluation, including the upper dental crown using coronal computed tomography, and treatment should be initiated promptly.

## Introduction

Brain abscess is a pyogenic disease secondary to a bacterial, tuberculous, fungal, or protozoan infection in the brain. Otitis media, tooth root infection, pneumonia, and sepsis are among the identified causes. Brain abscesses require urgent medical attention; thus, early detection and treatment are essential. Infection prevention is vital, and interventions such as maintaining oral hygiene, hand washing, and immunization are considered efficacious. Furthermore, early treatment of infection is also crucial [1-4]. Herein, we report a case of a patient diagnosed with a linear fracture of the frontal sinus four years prior. Even after the fracture had healed, inflammation from dental sinusitis advanced into the cranium, resulting in a brain abscess.

## Case presentation

**Figure 1:**
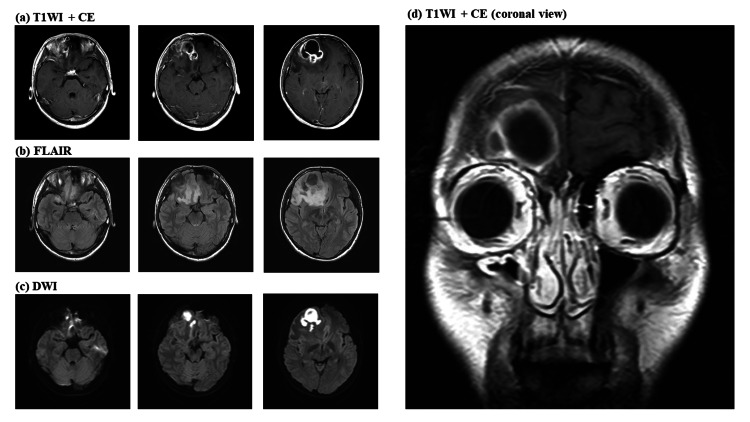
Initial MRI images of the patient who presented with a severe headache. (a) A ring-enhanced brain abscess measuring 40 mm was detected adjacent to the right frontal sinus on T1WI with contrast enhancement. (b) The brain abscess showed a mild right-to-left deviation with extensive FLAIR high signal around the lesion. (c) The brain abscess displayed a clear high signal on DWI. (d) Coronal T1WI with contrast enhancement revealed slight contrast findings at the site of the previous linear fracture. T1WI: T1-weighted image; CE: Contrast enhancement; FLAIR: Fluid-attenuated inversion recovery; DWI: Diffusion-weighted imaging.

A 21-year-old man complained of persistent frontal headache and fever for two weeks. He was generally healthy but had tooth decay with self-interrupted treatment in the right upper teeth. He is a non-smoker and an occasional alcoholic beverage drinker. For his educational attainment, he finished high school and is working as an electrical mechanic. He suffered from drug-induced urticaria at 10 years of age. He was diagnosed with sinusitis two weeks before admission, and antibiotic treatment was initiated. However, due to the persistence and progression of headache and fever, a head MRI was performed (Figure 1) during admission, which revealed a 40-mm-diameter right frontal brain abscess adjacent to the right frontal sinus on enhanced T1-weighted imaging.

**Figure 2:**
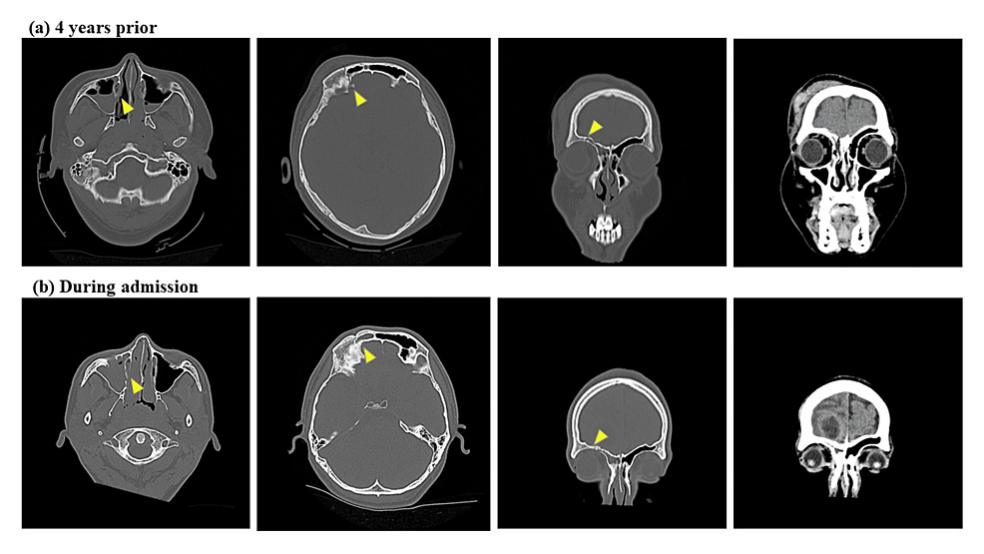
CT images of the frontal skull fracture four years prior and the brain abscess during this admission. (a) CT following a vehicular accident four years prior, showing a skull fracture in the right frontal and maxillary sinus. (b) CT taken during this admission due to a brain abscess, revealing incomplete fusion of the previous skull fracture in the right frontal and maxillary sinus.

He gave a history of trauma for frontal bone linear fracture, including the right frontal and maxillary sinus, with loss of consciousness. He was hospitalized for one week due to a vehicular accident four years ago. CT scan also showed an incomplete fusion of the previous skull fracture (Figure 2).

On the day of admission, he presented with somnolence and had a Glasgow Coma Scale score of 12-E3 V4 M5. No abnormalities were observed in the cranial nerves and limbs, and there were no cerebellar symptoms or neck stiffness. However, tenderness was noted in the frontal region upon tapping. Blood tests revealed a mildly elevated C-reactive protein and an increased WBC count (Table 1).

**Table 1:** Laboratory test results.

	Value	Normal range
Albumin	3.8 g/dL	3.8-5.3
Blood urea nitrogen	8 mg/dL	8-20
Creatinine	0.52 mg/dL	0.6-1.2
Sodium	134 mEq/L	134-148
Potassium	4.8 mEq/L	3.6-5.0
Aspartate aminotransferase	18 IU/L	0-40
Alanine aminotransferase	20 IU/L	0-35
γ-glutamyl transpeptidase	34 IU/L	0-40
C-reactive protein	1.55 mg/dL	0-0.3
White blood cell	14,100/µL	4,000-9,000
Hemoglobin	14.5 g/dL	13.0-16.0
Platelet	331,000/µL	130,000-300,000

Two days after hospitalization, aspiration of the right frontal brain abscess was performed, through a small craniotomy from the lateral side, avoiding past fracture sites. Cultures were taken during the procedure. Intraoperative findings revealed fracture lines from a prior injury. After the drainage surgery, the patient was administered an anticonvulsant and empiric therapy with three antibiotics. This treatment is planned to continue for over eight weeks, in line with the standard treatment period [5], followed by a gradual de-escalation (Table 2).

**Table 2:** Antibiotic treatment course. POD: Post-operative day; MEPM; Meropenem; VCM: Vancomycin; MNZ: Metronidazole; CTRX: Ceftriaxone; ABPC: Aminobenzylpenicillin; LVFX: Levofloxacin; MRSA: Methicillin-resistant Staphylococcus aureus.

POD	Antibiotics	Reason for selection
1-3	MEPM 2 g q8h + VCM 1g q8h + MNZ 500 mg q 6h	Empiric therapy
4-9	CTRX 2 g q12h + VCM 1g q8h + MNZ 500 mg q 6h	De-escalation
10-14	CTRX 2 g q12h + VCM 1g q 8h	MRSA negative, anaerobic bacteria coverage
15-16	CTRX 2 g q12h + MNZ 500 mg q 6h	Hepatic disorder, leukopenia
17-21	ABPC 2 g q4h + MNZ 500 mg q 6h	Persistence of leukopenia, anaerobic bacterial coverage ends
22-26	ABPC 2 g q4h	Drug eruption
27-77	LVFX 500 mg q24h	Oral medication

**Figure 3:**
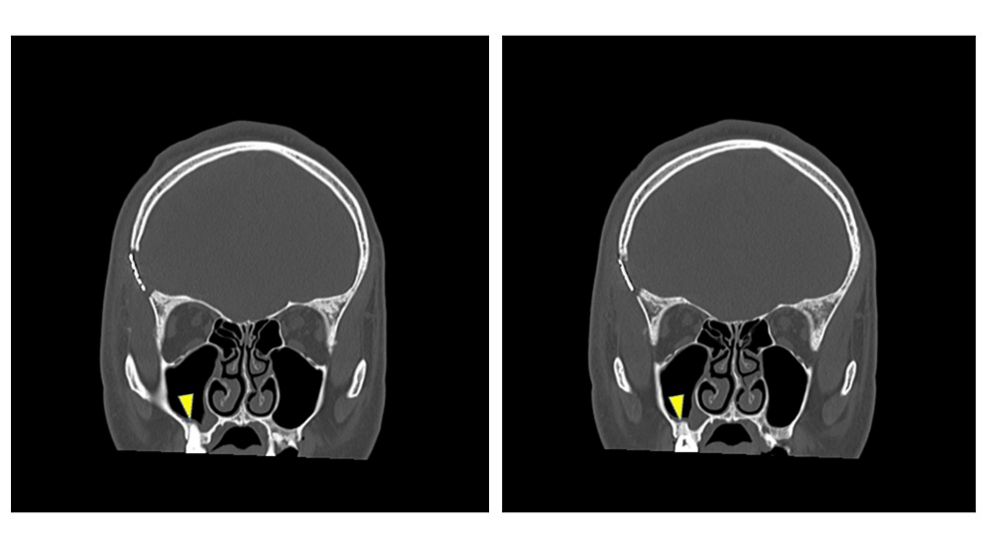
Coronal section including the upper dentition on the follow-up CT. A tooth with root inflammation showed osteolysis and was entrenched in the maxillary sinus.

He was persistently febrile (39°C) from the day after surgery to postoperative day (POD) 20. On POD 10, Streptococcus constellatus/milleri and Eikenella corrodens were detected in the culture; thus, antibiotic treatment was de-escalated based on drug sensitivity. For the right maxillary and frontal sinusitis, symptoms resolved on POD 11 shortly after antibiotic therapy was started. On follow-up images, residual abscess and perifocal edema gradually improved. On POD 19, generalized convulsive seizures occurred, and antiepileptic drugs were re-administered, with resolution of the seizures after. On POD 23, a bone defect at the bottom of the right maxillary sinus was found on the follow-up CT, which included the upper dental crown. Furthermore, bone translucency around the maxillary teeth was also observed (Figure 3).

On POD 28, to address the root inflammation, the patient underwent dental extraction, which was considered the cause of the dental sinusitis. Finally, antibiotic therapy was continued for more than eight weeks until the high signal on diffusion-weighted imaging was almost resolved. The results of the Wechsler Adult Intelligence Scale-III showed a verbal intelligence quotient (IQ) score of 70, a performance IQ score of 82, a full-scale IQ score of 73, a verbal comprehension score of 78, a perceptual organization score of 91, a working memory score of 74, and processing speed score of 72. The patient was discharged from the hospital with no neurological sequelae. There was no recurrence of seizures, so the antiepileptic drug was discontinued on POD 39. The electroencephalogram showed no abnormality on POD 43, and the occurrence of the generalized seizure was considered as an early postoperative seizure.

## Discussion

When antimicrobial agents are rendered ineffective, surgical treatment of brain abscesses should be aggressively considered, especially when the diameter of the abscess is >25 mm, the abscess is located near the ventricles and perforation is expected, or the risk of brain herniation is high due to the mass effect caused by the abscess. Regarding antibiotics, empirical antimicrobial therapy consisting of meropenem, vancomycin, and metronidazole was administered in line with standard treatment [1]. Our case was managed accordingly. The standard duration for antibiotic treatment is eight weeks [1]. However, in our case, the high signal on the MRI DWI persisted at eight weeks. Therefore, antibiotic treatment was extended to 11 weeks, at which point the high DWI signal subsided. This extension was due to concerns about a potential relapse if treatment was interrupted prematurely. Further debate will be needed on the optimal duration of antibiotic treatment. In a previous report, 205 brain abscesses were retrospectively reviewed for risk factors presenting with seizures; 48 patients had seizures, 17% were acute symptomatic seizures and 6.4% were unprovoked seizures. Risk factors highlighted included involvement of the frontal-parietal lobe and underlying valvular disease [6]. Our patient had been on antiepileptic drugs from the beginning, but they were withdrawn due to the absence of postoperative seizures, improvement in brain swelling, and the onset of leukaemia as a side effect of the drugs. It was during this period that she experienced her first seizure. Given that brain abscesses are predisposed to inducing seizures, a switch to another drug, while being attentive to potential drug-related side effects, might have been a viable option.

In this case, the patient had sustained a linear fracture in the right frontal sinus four years earlier. This fracture was near the location of the brain abscess, which was likely caused by untreated dental sinusitis from a right upper molar tooth. Even after the linear fracture had healed, the defense mechanism for infection was incomplete, which led to the development of the brain abscess. Since no other underlying disease was identified as the cause of the brain abscess and the fact that the brain abscess was secondary to bacteria indigenous to the nasal cavity, it was suggested that a direct spillover of sinusitis caused the brain abscess due to the structural deformity secondary to the frontal linear fracture from four years prior. This incomplete fusion led the patient to be susceptible to infection. Cases of brain abscess caused by dental peri-implantitis or dental infection were reported [7-9], and a case of delayed brain abscess following a transorbital perforating injury has been reported [10]. However, this case represents a rare instance illustrating a brain abscess resulting from dental sinusitis due to the incomplete fusion of a linear skull fracture. In such patients, immediate treatment of a dental infection is warranted. In the present case, a diagnosis of dental sinusitis was established by performing follow-up CT images, including the upper molars, with resolution of symptoms upon treatment. During CT imaging, the teeth are often excluded; however, taking a thorough image via a coronal section, including the upper teeth, should be employed for cases of brain abscess.

## Conclusions

Even if a frontal sinus linear fracture achieved fusion, left untreated root inflammation in the upper dentition can result in odontogenic sinusitis, and a brain abscess could develop secondary to dental sinusitis due to an incomplete infection defense mechanism. So, root infection of the tooth or sinus cavity should be treated immediately. Coronal CT, including the upper dental crown, should be conducted for root and sinus infections to employ immediate treatment.
